# Dynamic Progression of Hypertension and Diabetes in the Democratic Republic of Congo from 2019 to 2023

**DOI:** 10.3390/jcm13185488

**Published:** 2024-09-16

**Authors:** Karl B. Angendu, Pierre Z. Akilimali, Nguyen Toan Tran, Julien Magne

**Affiliations:** 1 Inserm U1094, IRD UMR270, CHU Limoges, EpiMaCT—Epidemiology of Chronic Diseases in Tropical Zone, Institute of Epidemiology and Tropical Neurology, OmegaHealth, University of Limoges, 87000 Limoges, France; karl.angendu_baki@unilim.fr (K.B.A.); julien.magne@unilim.fr (J.M.); 2 The Democratic Republic of Congo National Public Health Institute, Kinshasa P.O. Box 11850, Democratic Republic of the Congo; 3 Faculty of Medicine, Christian University of Kinshasa, Kinshasa P.O. Box 11850, Democratic Republic of the Congo; 4 Department of Nutrition, Kinshasa School of Public Health, University of Kinshasa, Kinshasa P.O. Box 11850, Democratic Republic of the Congo; 5 Patrick Kayembe Research Center, Kinshasa School of Public Health, University of Kinshasa, Kinshasa P.O. Box 11850, Democratic Republic of the Congo; 6 Australian Center for Public and Population Health Research, Faculty of Health, University of Technology, P.O. Box 123, Sydney, NSW 2007, Australia; nguyentoan.tran@uts.edu.au; 7 Faculty of Medicine, University of Geneva, Rue Michel-Servet 1, 1206 Genève, Switzerland

**Keywords:** arterial hypertension, diabetes, non-communicable disease, trends, Democratic Republic of the Congo

## Abstract

**Introduction:** The Democratic Republic of Congo (DRC) does not have national prevalence data for arterial hypertension (HTN) or diabetes (type I and II combined) to aid evidence-based decision-making, despite the assumption of epidemiological transition in low- and middle-income countries. The aim of this study was to estimate a proxy of prevalence for HTN and diabetes. **Methodology:** This study used routine monthly reported data pertaining to HTN and diabetes from the District Health Information Software 2 (DHIS2), spanning 2019–2023. Data underwent quality assessment and adjustments using standardization before analysis. Equity analyses were carried out at the national and sub-national levels. Epidemiological curves and maps were produced to analyze trends in the prevalence of HTN and diabetes among adults aged 18 and over. Permission to use the data was obtained from the regulatory authority. **Results:** Over five years, incidence of HTN increased from 13.23% (CI 95%: 13.22–13.24) to 15.23% (CI 95%: 15.22–15.24) (+15.1% relative increase), and diabetes rose from 2.73% (CI 95%: 2.72–2.74) to 3.345% (CI 95%: 3.34–3.35) (+16.3% relative increase), with provincial variations observed. **Conclusions:** In the DRC, hypertension and diabetes are advancing rapidly. Primary and preventative healthcare services and public health interventions must prioritize these diseases.

## 1. Introduction

Despite continuous economic growth in the Democratic Republic of the Congo (DRC) in recent years, it consistently ranks amongst countries with the worst health indicators globally. The DRC ranks among the five lowest-income countries globally; by 2023, over 74.6% of the population of the Congo were living below the poverty line, with a daily income of less than USD 2.15 [1]. Conflict and instability have negatively impacted the government’s ability to translate the DRC’s economic growth into the provision of basic health services for its citizens [2]. The rapid process of urbanization, along with expanding economies and population expansion, has resulted in an increase in the number of middle-income households in Africa. Consequently, more Africans, particularly those in the DRC, have changed their consumption patterns and lifestyles. Similar to trends in more developed nations, this has resulted in a rise in overweight and obesity rates throughout Africa, greatly elevating the likelihood of developing diabetes and high blood pressure [3,4,5].

Urbanization and changing lifestyles are characterized by the growing prominence of modern merchants, including supermarkets and fast-food restaurants [6,7,8]. The expansion of supermarkets is associated with the increased consumption of processed foods and increased body mass index (BMI) [9,10]. The primary drivers of food system modernization are increased wealth and globalization [11,12,13,14]. Current research indicates that the modernization of food retail can potentially lower the cost of calories for urban consumers. However, it may also contribute to a shift in dietary patterns toward highly processed foods, which are deficient in the components necessary for a healthy diet [15,16].

Epidemiology in the DRC remains focused on communicable diseases (CDs) despite the hypothesis of an epidemiological transition taking place in tropical zones [17]. The most recent stepwise surveys in Kinshasa were conducted in 2008, finding that 15.2% of the population were hypertensive and 14.2% were diabetic [18]. However, both precise and estimated data on the prevalence of hypertension (HTN) and diabetes are still lacking. This information gap hinders effective strategizing and the implementation of initiatives to address these chronic diseases. Our study analyzed the evolution of HTN and diabetes in the DRC over the last five years (from 2019 to 2023) to provide evidence for healthcare decision-making and policy.

## 2. Methodology

This study contributes to the existing literature by using facilities data (DHIS2) from 2019 to 2023 and a proxy method to assess the extent and trend of the two most prevalent chronic non-communicable diseases in the DRC: hypertension and diabetes.

### 2.1. Source of the Data

The data source used was District Health Information Software 2 (DHIS2). Healthcare facilities (HFs) submit monthly HTN and diabetes data to the health district or health zone (HZ) offices using standardized reports. The HZs enter the data on computers running the latest version of the District Health Information System Software, commonly known as DHIS2. HZ files are collated at the national and provincial levels and assessed for completeness and quality. At the HZ level, problems of incomplete information, outliers, and data consistency are initially detected and rectified, but these issues can still affect the reliability of HF data. To overcome these problems, we used a set of approaches established by the World Health Organization (WHO) [19] and the Countdown to 2030 project, also used in a similar study [20]. This analysis focuses on routine HTN, and diabetes cases reported by health facilities in the DHIS2 system. Data were extracted in January 2024 for each month between January 2019 and December 2023. The dataset is a census dataset representing all facilities reporting to the DHIS2. The data set also exhibits a facility-based bias because some facilities do not report in the DHIS2 platform.

### 2.2. Data Quality Assessments

We organized the DHIS2 data in standardized Excel sheets. Monthly totals were collected from January 2019 to December 2023 for various interventions in different HZs, including data on diabetes and HTN notifications, data related to antenatal and delivery care, immunization and outpatient visits. We also extracted additional data on the monthly completeness rate of the HF reports. The analysis started with evaluating the quality of the data and creating refined datasets (Supplementary Figure S1). This involved addressing incomplete reporting by HFs and rectifying missing values and extreme outliers in the monthly data supplied by each HZ. The evaluation and modifications were conducted using standard protocols.

Quality assessments were conducted on the zonal, provincial, and national data. The HZs exhibiting problematic reporting rates and inconsistencies were identified for additional scrutiny and, if necessary, rectification. Inconsistency was measured using four indicators chosen because they are available in all HFs, including the ratio between the first-trimester antenatal consultation rate and the immunization coverage rate for the first dose of pentavalent vaccine. It also included the ratio between the immunization coverage rate for the first dose of pentavalent vaccine and the immunization coverage rate for the third dose of the same vaccine. Consistency was defined as the value of the ratio between 1.05 and 1.5. The zonal-level assessment evaluated the impact of HF reporting completeness on the number of reported occurrences. Reporting completeness refers to the proportion of facilities that submitted data for a specific month out of all the facilities that were required to do so. We compiled the percentage of district-months with facility reporting rates below 90% and provided a list of all district-months with facility reporting rates below 75%. To account for partial reporting in all other HZs, we considered the extent to which HFs reported data and the predicted level of service provision from facilities that did not.

### 2.3. Data Adjustment

In the latter’s case, we employed an adjustment factor that varied between 0 and 1. A value of 1 indicates that the level of services given by non-reporting facilities is similar to that of reporting facilities, while a value of 0 assumes that non-reporting facilities offer no services. The understanding of the delivery of services and the allocation of HFs in the country is influenced by the determination of this adjustment factor. An adjustment factor of 0.75 was used following deliberation among the DHIS2 team to determine the most suitable factor for each intervention. Additionally, it was assumed that no services were offered in HZs situated in unstable areas. If there were any missing numbers, the median value of the calendar year was used unless there was evidence suggesting that it was an actual zero. Extreme outliers were detected by calculating a modified Z-score, a standardized measure of how much an observation deviates from the median.

This was achieved by dividing the difference between the observation and the median by the median absolute deviation. Extreme outliers were defined as monthly data points with scores higher than five standard deviations from the annual median. These were rectified by substituting a value derived from the median value of the calendar year. The summary was generated using a bottom-up approach, utilizing the cleaned and corrected HZ data. Additionally, we generated summaries based on geographical regions.

### 2.4. Analytical Approaches

To determine the estimated proportions of HTN and diabetes among the population of individuals ≥ 18 years old, we used the adjusted number of HTN, and diabetes extracted from DHIS2 as the numerator, which was multiplied by the Incidence–Prevalence Ratio (IPR), identified in this paper as “sigma factor”. The IPR is a proposed metric for defining epidemic control. IPR provides a comprehensive view of an epidemic’s dynamics. By leveraging these two measures, health authorities can assess the rate of new cases relative to the overall number of HTN patients. IPR for HTN, as a chronic disease for a specific year, is the ratio between the incidence and prevalence of HTN [21]. The most updated study published for the DRC reported a prevalence ratio of 29.0% and an incidence rate of 2.4%, which gave an IPR of 0.08 [22].

This corrector factor was applied for HTN and diabetes cases, presuming that this ratio will remain consistent over time in the absence of any intervening factors affecting the progression of the two diseases. The denominator was 45% (proportion of individuals 18 years of age and older) of the whole population [23]. The estimated percentage used as a proxy of prevalence was calculated by year and province. The prevalence proxy utilized in this study enabled us to include demographic variations among provinces when comparing the temporal trends of HTN and diabetes. Two summary metrics were utilized to illustrate the disparity in regional inequality at the subnational level: the Mean Absolute Difference to the Mean (MADM) and the Mean Relative Difference to the Mean (MRDM). The two estimated proportions (diabetes and AHT) were utilized to compare trends over time. The generated maps also enabled us to compare trends by province and year. QGIS 3.41.3 and STATA 17 (Stata Corp, College Station, TX, USA) software were used.

### 2.5. Ethical and Legal Aspects

To ensure confidentiality, we identified only those variables guaranteeing anonymity in the database. Regarding informed consent, we did not have any contact with the patients, so no biological procedures were used in the collection or processing of the data. The use of this study’s results is strictly limited to exploitation related to its objectives, and the authors have reported no conflicts of interest.

### 2.6. Patient and Public Involvement

Patients and the public were not involved in the study design, development of the research questions, recruitment into or conduct of the study, or definition of the outcome measures. The results were not distributed to the participants themselves.

## 3. Results

### 3.1. Data Quality Assessment

The overview of data quality includes the national values for each year from 2019 to 2023, as well as the percentage of HZs with high-quality score data for each of the three characteristics of data quality (see Figure S1 and Table S1). This table of data quality assessment scores shows that the data quality in DHIS2 improves over time. Completeness improved progressively from 2019 to 2023, reaching at least 95% for four indicators that were selected on the basis of their high availability in healthcare facilities: antenatal care (ANC), deliveries in a health facility, outpatient or ambulatory consultations, and childhood immunization. Extreme outliers were less than 10% for each of the five years, and consistency was good, as the ratios of the various indicators were between 1.05 and 1.5, and the percentage of HZs with low reporting rates (less than 90%) decreased over time, except for hospital admissions (Figure S2).

### 3.2. Arterial Hypertension and Diabetes Trends

**Figure 1:**
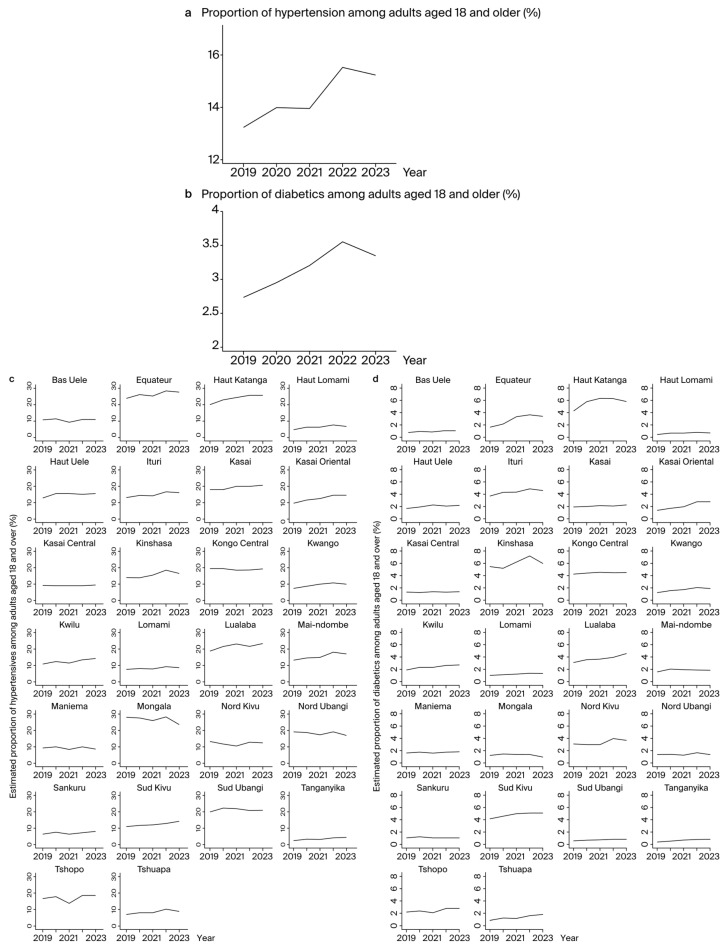
Estimated proportion of hypertensives and diabetics among adults aged 18 years and over: (**a**) Proportion of hypertensives among adults aged 18 years and over in the Democratic Republic of Congo. (**b**) Proportion of diabetics among adults aged 18 years and over in the Democratic Republic of Congo. (**c**) Proportion of hypertensives among adults aged 18 years and over in provinces. (**d**) Proportion of diabetics among adults aged 18 years and over in provinces.

The trends in HTN and diabetes are shown in Figure 1. The evolution of HTN in the DRC over the five years is reported in Figure 1a and demonstrates a growing trend. The proportions of HTN increased from 13.23% (CI 95%: 13.22–13.24) in 2019 to 15.23% (CI 95%: 15.22–15.24) in 2023, representing an absolute increase of +2% and a relative increase of +15.1%. The diabetes trend steadily increased over the first four years, with a peak in proportion in 2022 (3.55%) before a slight decline in 2023 (Figure 1b). The proportion of diabetes increased from 2.73% (CI 95%: 2.72–2.74) in 2019 to 3.345% (CI 95%: 3.340–3.35) in 2023, representing an absolute increase of +0.61% and a relative increase of +16.3%. Provincial trends varied for both HTN and diabetes.

At the provincial level (Figure 1c), among the 26 provinces, the five highest proportions of HTN were in Haut Katanga, Kasai, Lualaba, Mongala, and Nord Ubangi. In Haut Katanga, the proportion increased from 20% in 2019 to 25.9% in 2023, representing an absolute increase of +5.9% and a relative increase of +29.5%. In Kasai, the proportion increased from 17.9% in 2019 to 20.5% in 2023, representing an absolute increase of +2.6% and a relative increase of +14.5%. In Lualaba, the proportion increased from 18.5% in 2019 to 23.3% in 2023, an absolute increase of 4.8% and a relative increase of 25.9%. In Mongala, the proportion was 28% in 2019 and 23.5% in 2023, representing an absolute decline of 4.5% and a relative decline of 16%. In Nord Ubangi, the proportion was 19.3% in 2019 and 17% in 2023, representing an absolute decline of 2.3% and a relative decline of 11.9%.

Regarding diabetes (Figure 1d), the highest proportions were in Haut Katanga, Kinshasa, and Lualaba. In Haut Katanga, the proportion increased from 4.1% in 2019 to 5.7% in 2023, representing an absolute increase of 1.6% and a relative increase of 39%. In Kinshasa, the proportion increased from 5.5% in 2019 to 5.9% in 2023, representing an absolute increase of 0.4% and a relative increase of 7.3%. In Lualaba, the proportion increased from 3% in 2019 to 4.5% in 2023, or an absolute increase of 1.5% and a relative increase of 50%. No provinces showed a decline in diabetes prevalence from 2019 to 2023.

**Figure 2:**
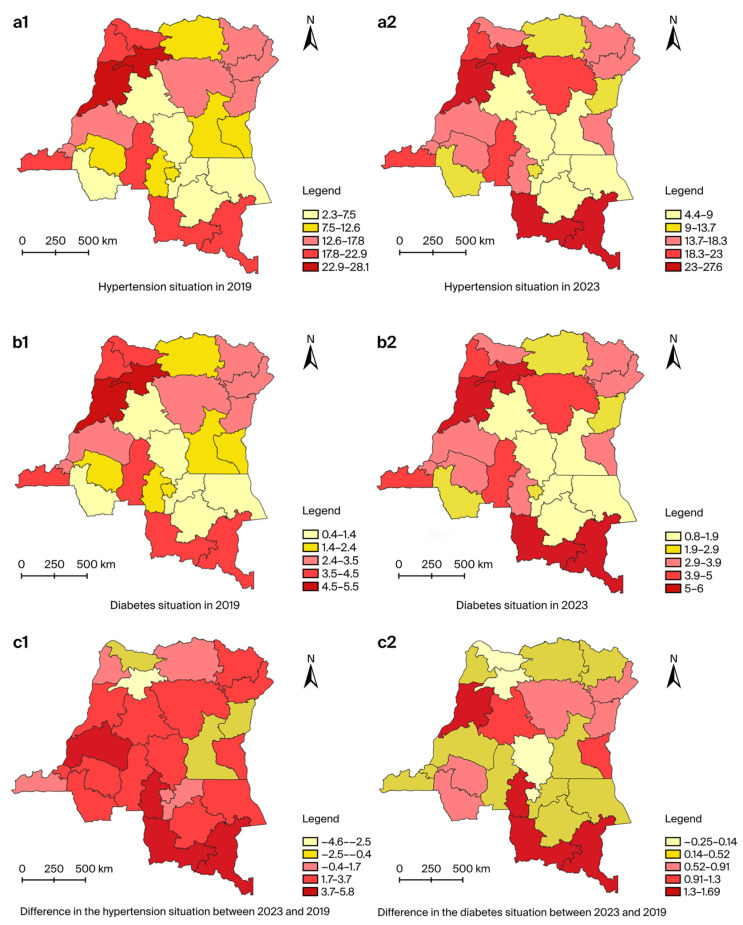
Change in the estimated proportions of hypertensives and diabetics among adults aged 18 years and over from 2019 to 2023: (**a**) Change in the estimated proportions of hypertensives among adults aged 18 years and over from 2019 to 2023: (**a1**) hypertension situation in 2019; (**a2**) hypertension situation in 2023; (**c1**) difference in the hypertension situation between 2023 and 2019. (**b**) Change in the estimated proportions of diabetics among adults aged 18 years and over from 2019 to 2023: (**b1**) diabetes situation in 2019; (**b2**) diabetes situation in 2023; (**c2**) difference in the diabetes situation between 2023 and 2019.

The overall trends for HTN and diabetes are also depicted in maps (Figure 2).

### 3.3. Equity Analysis

**Figure 3:**
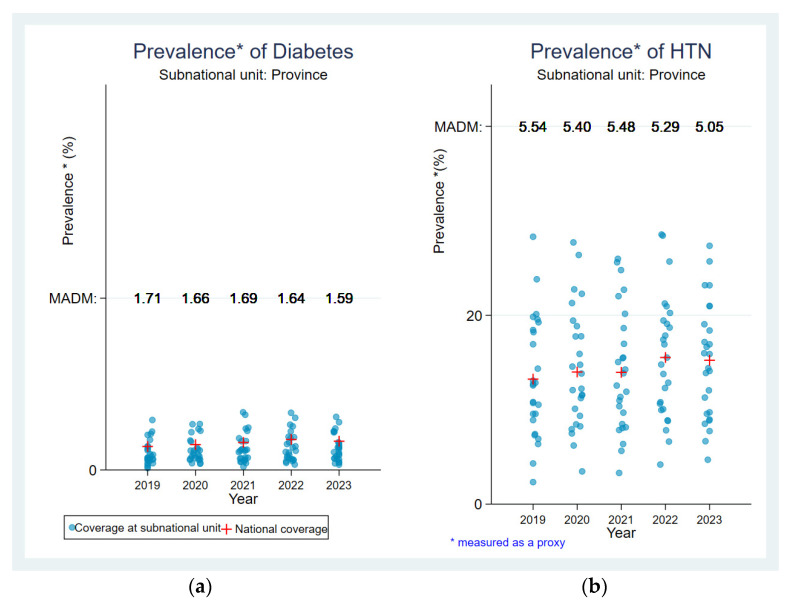
Equity analysis for the estimated proportion of hypertension and diabetes: (**a**) Equity analysis for the estimated proportion of hypertension. (**b**) Equity analysis for the estimated proportion of diabetes.

On the basis of the point dispersion and MADM values (Figure 3), an assessment of equity between the different provinces was carried out over the five years. There was an inequality in the proportions between provinces for HTN (Figure 3). There was little inequality in the proportions between provinces for diabetes (Figure 3). For HTN, high inequalities were observed in all five years. However, in relation to diabetes, they were less marked in all five years.

## 4. Discussion

Our study found that the proxy prevalence for HTN increased by 15.1% over the five years, rising from 13.23% (CI 95%: 13.22–13.24) in 2019 to 15.23% (CI 95%: 15.22–15.24) in 2023. This rate of progression is similar to that observed in a recent study from Sud Kivu province, where the prevalence increased by 16.9% over eight years [16]. This is likely due to our study encompassing the whole country. Similarly, the diabetes prevalence proxy increased by 16.3% over the five years, from 2.73% (CI 95%: 2.72–2.74) in 2019 to 3.345% (CI 95%: 3.340–3.35) in 2023. To the best of our knowledge, no study in the DRC has investigated the evolution of diabetes over two different time periods. However, in a comparative study conducted in Mozambique, the prevalence of diabetes more than doubled in 11 years from 2.9% to 7.4% [24].

This research is the first to estimate a proxy for the national prevalence of HTN and diabetes in the DRC, using routine data. It is also the first study to have followed the evolution of HTN and diabetes on a national scale over five years. However, other studies have analyzed the evolution of the prevalence of HTN between two periods [22,25,26,27]. Our study provides evidence for the progression of NCDs in general, specifically HTN and diabetes in the DRC. Several studies point to an epidemiological transition taking place in low- and middle-income countries [17]. This study contributes to understanding the rapid progression of NCDs in these countries, specifically in the DRC. HTN affects over one in four individuals in just five years, consistent with projections anticipating a doubling of hypertensive cases in sub-Saharan Africa by 2030 [28]. Similarly, diabetes prevalence has increased significantly, affecting one in six people over the same period, aligning with forecasts of a 129% increase in diabetes cases globally in 25 years [29].

One limitation of our study was that it did not compare the progression of HTN and diabetes with the evolution of communicable diseases over the same period to better understand the dynamics of NCDs. In the present research, a more detailed study of the difference in the progression of HTN and diabetes in the different provinces of the DRC was not carried out. It focused mainly on the overall progression at national level. Future research should also investigate the factors associated with the rapid progression of these disease groups in different provinces. Our current findings indicating the rapid evolution of HTN and diabetes are important for policymakers. Many low- and middle-income countries lack regulatory frameworks for NCDs, making these results crucial for guiding the development of standards, guidelines, and public health strategies aimed at reducing NCD-related morbidity and mortality [30]. Clinicians should also take heed, emphasizing early detection, management, and patient education on preventing and managing HTN and diabetes. Our results should serve as a basis for guiding the development of standards, guidelines, procedures, and plans with the ultimate aim of reducing the burden of morbidity and mortality associated with NCDs. Public health initiatives should integrate NCD-related issues to effectively address the growing burden. These initiatives should promote the identification of at-risk populations, targeted prevention actions, the fight against sedentary lifestyles, nutritional education, access to care, and screening, given that cardiovascular and metabolic disorders often evolve asymptomatically.

In our study, an innovative methodological approach was used to adjust the reported cases of HTN and diabetes via DHIS2. The approach consisted of dividing the notified cases of these two diseases by the quotient of the incidence/prevalence ratio of HTN. This approach could be used for studies wishing to estimate a proxy of the prevalence of NCDs in the context of low- and middle-income countries, where financial resources are very limited for implementing research projects requiring field data collection at the national level. The estimates given in this study closely align with or resemble the newly published estimates from the latest DHS 2023 survey in the DRC, proposing that utilizing DHIS2 data can function as a substitute for estimating the occurrence of diabetes and hypertension at the national level, with inadequate resources for generating population-level estimates. Indeed, the current study reveals that 15.2% of the population suffered from hypertension in 2023. The DHS survey 2023 indicates that 14% of men between the ages of 15 and 49 have been categorized as hypertensive [31]. This study indicates that the prevalence of diabetes in the population was 3.3% in 2023 while the DHS indicates that 4.0% of women and men between the ages of 15 and 49 had diabetes [31]. This demonstrates that this approach can be utilized to obtain approximate assessments of the extent of hypertension and diabetes from DHIS2 data, particularly in situations where DHS or STEPWISE data is not available. The most recent STEPWISE study conducted in the Democratic Republic of Congo (DRC) was in 2008, and it only focused on the city of Kinshasa.

Data collection itself was not carried out in this study; aggregated DHIS2 data were used, which were missing some important variables such as age, sex, and classification of HTN and diabetes according to type and stage. DHIS2 contains only information collected in the HFs integrated into this system. This could have led to selection bias. However, the use of a national database containing data collected in all the HZs of the DRC is a strength. Although some HFs are not integrated into the DHIS2, the majority are and are publicly available at very affordable rates, thus encouraging demand. It is also true that COVID-19 has swept the world in general, but the impact has varied from one geographical area to another. In the DRC, it really hit home in the first six months, from March to August 2020. This was mainly in Kinshasa, but not in the provinces. During the same period, awareness-raising was carried out to encourage the population to continue frequenting HFs. Efforts have also been made by the government to ensure that there are no disruptions to the usual services. This would have helped to maintain the completeness of the data. During the assessment of data quality prior to statistical analysis, an improvement in completeness was observed in 2020 and 2021, compared with 2019, the year in which there was no COVID-19.

The methodology employed in this study must undergo validation by utilizing data from nations that have comprehensive stepwise survey data coverage in countries where the population size is accurately determined through a census. The DRC lacks accurate knowledge of its population size, as the most recent census was carried out more than three decades ago. The accuracy of the population sizes of each province may have an impact on the estimations reported in this study at the provincial level. Further analysis is planned over the next years, using the same methodological approach, to estimate prevalence proxies in a non-COVID-19 context. The absence of data from this study about socioeconomic status, biochemical parameters and other factors that could affect HTN and diabetes trends, such as genetics, lifestyle, and literacy level, is another limitation of this study. 

## 5. Conclusions

In the DRC, HTN and diabetes are rapidly advancing, necessitating heightened surveillance and comprehensive primary and public health interventions. While communicable diseases currently receive significant attention, a holistic approach that integrates NCDs is crucial. Low- and middle-income countries like the DRC should explore modeling studies to forecast trends of HTN and diabetes, leveraging the routine data and innovative methodological approaches proposed in this study.

## Data Availability

The original contributions presented in the study are included in the article/Supplementary Material, further inquiries can be directed to the corresponding author.

## Supplementary Materials

The following supporting information can be downloaded at: https://www.mdpi.com/article/10.3390/jcm13185488/s1. Figure S1. Data analysis procedures. Table S1. Summary of the data quality for the reported healthcare facilities. Figure S2. Percentage of health zones with a low completeness rate (<90) by service and year.
