# Supplementary material for: Dynamic Progression of Hypertension and Diabetes in the Democratic Republic of Congo from 2019 to 2023
Source: J Clin Med. 2024 Sep 16;13(18):5488. doi: 10.3390/jcm13185488 (PMC11431946; doi:10.3390/jcm13185488)
Supplement: Supplementary file 1 [file jcm-13-05488-s001.zip › jcm-3138639-supplementary.pdf]

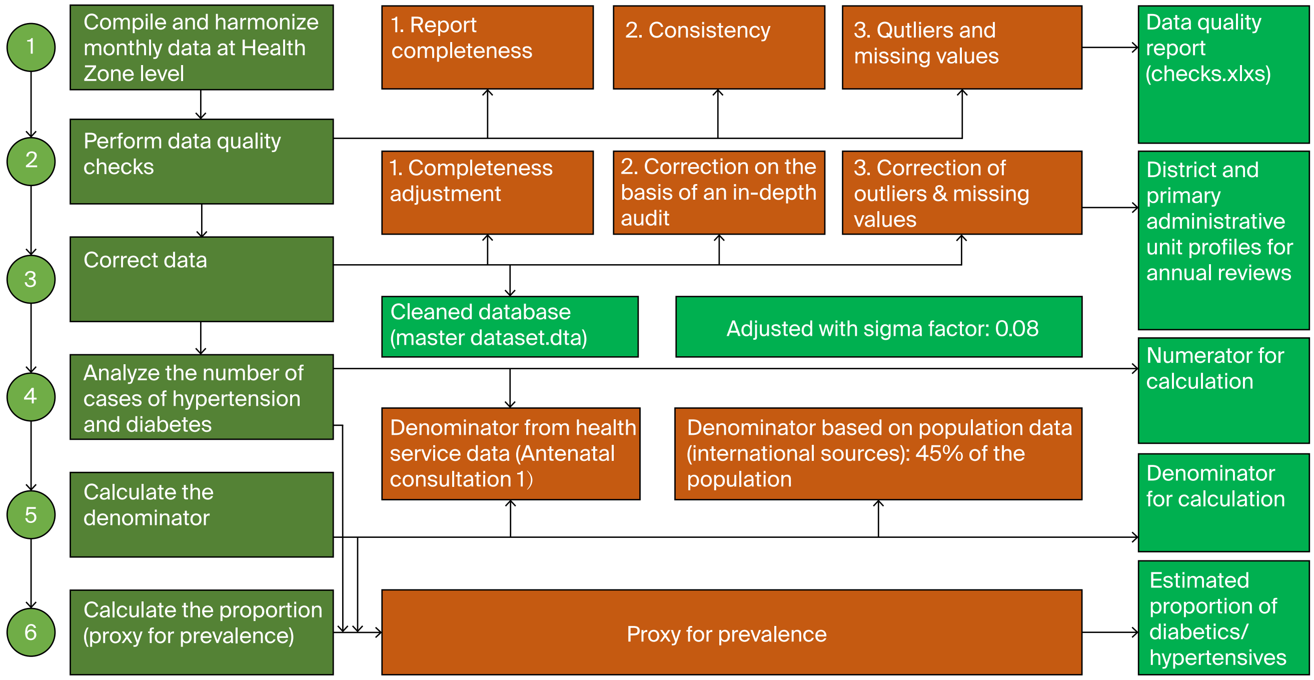

Figure S1. Data analysis procedures.

Table S1. Summary of the data quality for the reported healthcare facilities.

	Data Quality Indicators	2019	2020	2021	2022	2023
1	**Completeness of monthly reports from healthcare facilities (average of antenatal care (ANC), institutional deliveries, vaccinations, and outpatient department)**					
1a	% of monthly reports expected (national)	90	95	96	98	99
1b	% of the health zone (HZ) with complete healthcare facility (HC) reports ≥90%	68	82	82	92	95
1c	% of HZ with no missing values for the 4 forms	92	92	92	92	91
**2**	**Extreme outliers (average of antenatal consultations, deliveries, vaccinations, and outpatient visits)**					
2a	% of monthly values that are not extreme outliers (national)	95	97	96	95	93
2b	% of the HZ with no extreme outliers during the year	88	90	88	86	83
**3**	**Consistency of annual reports**					
3a	ANC1/penta1 ratio in the reported data (national)	1,07	1,07	1,16	1,22	1,17
3b	Penta1/penta3 ratio in the reported data (national)	1,07	1,06	1,06	1,07	1,07
3c	% of the HZ with an ANC1-penta1 ratio within the expected range	65	69	74	83	81
3d	% of the HZ with a penta1-penta3 ratio within the expected range	99	100	96	99	98
4	**Annual data quality score (average 1a, 1b, 2a, 2b, 3c, 3d)**	84	89	89	92	92

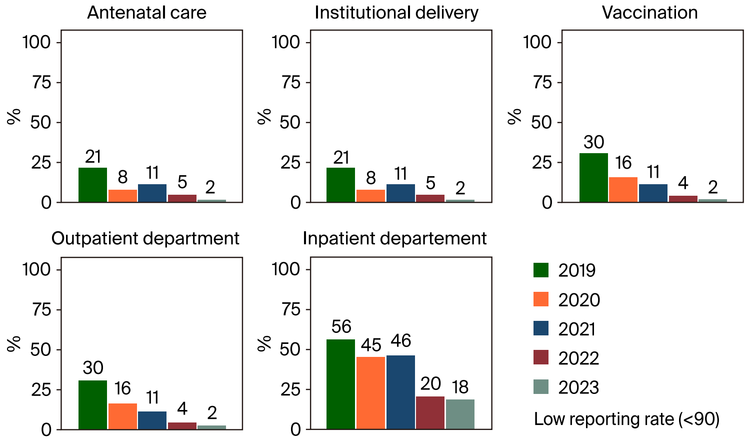

Figure S2. Percentage of health zones with a low completeness rate (<90) by service and year.
